# Coronavirus disease 2019 and acute cerebrovascular events: a comprehensive overview

**DOI:** 10.3389/fneur.2023.1216978

**Published:** 2023-06-28

**Authors:** Wanzhou Zhang, Li Ling, Jie Li, Yudi Li, Yajie Liu

**Affiliations:** ^1^Department of Neurology, Shenzhen Hospital, Southern Medical University, Shenzhen, Guangdong, China; ^2^The Third School of Clinical Medicine, Southern Medical University, Guangzhou, China

**Keywords:** COVID-19, SARS-CoV-2, hypercoagulability, ischemic stroke, mechanical thrombectomy

## Abstract

Since the Corona Virus Disease 2019 (COVID-19) pandemic, there has been increasing evidence that severe acute respiratory syndrome coronavirus 2 (SARS-CoV-2) infection is associated with acute cerebrovascular events such as cerebral infarction, cerebral hemorrhage, and cerebral venous thrombosis. Although the mechanism of cerebrovascular complications among COVID-19 patients has not been adequately elucidated, the hypercoagulable state, excessive inflammation and ACE-2-associated alterations in the renin-angiotensin-aldosterone system after SARS-CoV-2 infection probably play an essential role. In this overview, we discuss the possible mechanisms underlying the SARS-CoV-2 infection leading to acute cerebrovascular events and review the characteristics of COVID-19-related acute cerebrovascular events cases and treatment options available worldwide.

## 1. Introduction

Since December 2019, the coronavirus disease 2019 (COVID-19) pandemic, caused by severe acute respiratory syndrome coronavirus 2 (SARS-CoV-2), has infected billions of people worldwide, resulting in several million deaths (1). The common symptoms described by COVID-19 patients are dyspnea, fever, dry cough, arrhythmia, myalgia, and olfactory and gustatory dysfunction (2, 3). In addition to the symptoms commonly associated with viral infections, there are increasing reports showing an increased incidence of acute cerebrovascular events in COVID-19 patients (4, 5). Although the mechanisms underlying such complications are not fully understood, hyperinflammation, cytokine storm, hypercoagulable state, and cardiogenic embolism probably perform critical roles (6, 7). In this review, we investigate the potential mechanisms underlying acute cerebrovascular events resulting from SARS-CoV-2 infection. Furthermore, we review the characteristics of case reports involving cerebrovascular complications in COVID-19 patients, together with the treatment options.

## 2. Pathophysiological processes relating COVID-19 and cerebrovascular events

**Figure 1:**
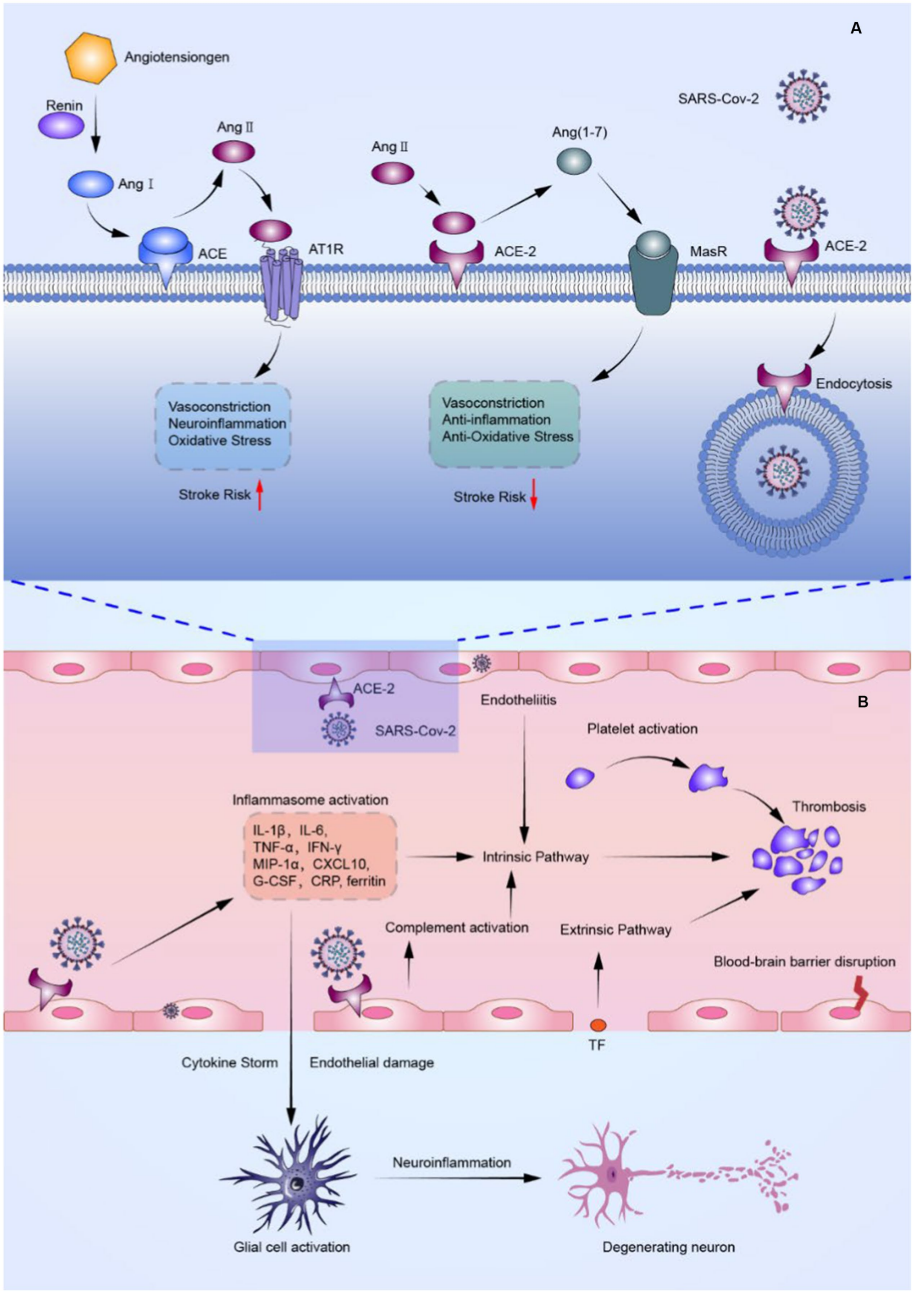
FIGURE 1 (Continued)The probable mechanism of ischemic stroke in COVID-19 patients. **(A)** Schematic overview of SARS-CoV-2 effects on the renin-angiotensin-aldosterone system (RAAS). SARS-CoV-2 not only binds to ACE-2 causing downregulation of ACE-2, but also competes with Ang II for binding to ACE-2, resulting in an increase in Ang II and a decrease in Ang (1–7). Such events result in abnormalities of the RAAS, causing vasoconstriction, inflammation, and thrombosis. **(B)** Schematic illustration of the inflammatory storm and thrombosis triggered by endothelial cell damage associated with SARS-CoV-2 infection. By binding to ACE-2, SARS-CoV-2 can directly invade cerebrovascular endothelial cells, causing endothelial damage and tissue factor exposure, as well as the release of cytokines such as IL-6 and TNF-, and multiple factors mentioned above activate the coagulation system, initiating intrinsic and extrinsic coagulation pathways and causing arterial and venous thrombosis. Furthermore, cytokine stimulation causes astrocytes and microglia to become hyperactive, resulting in a pro-inflammatory state that exacerbates neuroinflammation. ACE, angiotensin-converting enzyme; ACE-2, angiotensin-converting enzyme 2; Ang I, angiotensin I; Ang II, angiotensin II; Ang (1–7), angiotensin 1–7; AT1R, angiotensin II type-1 receptor; CRP, C-reactive protein; CXCL 10, CXC chemokine ligand-10; G-CSF, granulocyte colony-stimulating factor; IFN-γ, interferon-γ; IL-1β, Interleukin-1β; IL-6, interleukin-6; MasR, Mas receptor; MIP-1α, macrophage inflammatory protein 1α; TF, tissue factor; TNF-α, tumor necrosis factor-α.

Even though multiple SARS-CoV-2 vaccines have been produced, novel variants of SARS-CoV-2 have made COVID-19 a persistent epidemic that requires the attention of the medical community (8). Therefore, understanding probable pathophysiological mechanisms connecting cerebrovascular diseases with COVID-19 is imperative for effective therapy. A brief description of the possible mechanisms of ischemic stroke in COVID-19 patients is presented in Figure 1.

### 2.1. SARS-CoV-2 triggers endothelial inflammation

Spike glycoproteins that are present on SARS-CoV-2 outer envelope have a high affinity for human angiotensin-converting enzyme 2 (ACE-2) (9). ACE-2, a transmembrane receptor with enzymatic properties, is expressed in a variety of cells, including cardiomyocytes, vascular endothelial cells, and lung epithelial cells, among others (10, 11). The combination of SARS-CoV-2 and ACE-2 enables the virus to invade into cells.

Through its high affinity for ACE-2, SARS-CoV-2 may invade vascular endothelial cells directly, which leads to endothelial inflammation and ultimately thrombus formation. Autopsy samples from the lungs, heart, kidneys, and intestines have shown signs of endothelial inflammation and apoptosis after SARS-CoV-2 infection (12). Similar to these organs, SARS-CoV-2 can directly intrude into cerebrovascular endothelial cells by binding to ACE-2, thereby increasing the risk of vasculitis, blood–brain barrier (BBB) injury, and secondary stroke (13). In addition, cerebral vasculitis can lead to arterial remodeling, narrowing or dilatation, and fragility of the vessels, followed by ischemic or hemorrhagic stroke (14).

Vascular endothelial injury is not only attributed to direct invasion of vascular endothelial cells by SARS-CoV-2, but is also influenced by the body’s systemic immune response to the virus, the so-called cytokine storm. A variety of cytokines, including Interleukin-1β (IL-1β), interleukin-6 (IL-6), interferon-γ (IFN-γ), tumor necrosis factor-α (TNF-α), macrophage inflammatory protein 1α (MIP-1α), CXC chemokine ligand-10 (CXCL 10), granulocyte colony-stimulating factor (G-CSF), C-reactive protein (CRP), and ferritin were found to be markedly increased in COVID-19 patients, especially in those who were severely ill (15, 16). Excessive levels of pro-inflammatory cytokines may disrupt the integrity of tight junctions in BBB (17). Moreover, in the hyperinflammatory state of COVID-19, excessive production of acute inflammatory response proteins and adhesion molecules as well as circulating activated leukocytes may lead to an enhanced inflammatory process in the ischemic brain (18). Furthermore, astrocytes and microglia receive cytokine stimulation and become overactivated, shifting to a pro-inflammatory state, which exacerbates neuroinflammation (19). This may explain the increased severity of stroke observed in patients with COVID-19.

### 2.2. SARS-CoV-2 induced hypercoagulation

Hypercoagulable states may increase the incidence of ischemic stroke and cerebral venous thrombosis (CVT) (20). Several reports on the hypercoagulable state related to severe COVID-19 have been published that have attributed the hypercoagulable to endothelial cell injury, excessive inflammation, platelet activation, immobilization, dehydration, activated complement system, and hypoxia (21–23). SARS-CoV-2 directly invades endothelial cells and promotes the release of inflammatory factors, such as IL-6 and TNF-α, leading to endothelial cell damage and tissue factor (TF) exposure (11). In the course of COVID-19 disease, multiple factors mentioned above activate the coagulation system and initiate intrinsic and extrinsic coagulation pathways, causing arterial and venous thrombosis (6). In addition, immobilization in hospitalized patients, especially in severe cases, causes blood stagnation that significantly increases the incidence of deep vein thrombosis, pulmonary embolism and embolic stroke (24).

In studies of COVID-19-related cerebrovascular disease, elevated levels of coagulation-related indicators such as D-dimer, fibrinogen, antiphospholipid antibodies, and platelet count were detected (25, 26). D-dimer, a marker of endogenous fibrinolysis, is generated by the degradation of cross-linked fibrin with fibrinolytic enzymes during the last step of clot formation (27). A report showed substantially higher D-dimer levels in severe COVID-19 patients than in common COVID-19 patients (28). The study encompassing 191 individuals diagnosed with COVID-19 revealed that the levels of D-dimer exceeding 1 μg/mL were linked to heightened mortality (29). According to Yaghi et al., there was a higher prevalence of elevated D-dimer levels among COVID-19 patients who developed stroke. The authors proposed that a hypercoagulable state may be a contributing factor to the occurrence of stroke in COVID-19 patients (30).

### 2.3. SARS-CoV-2-related renin-angiotensin-aldosterone system alterations

As a component of the renin-angiotensin-aldosterone system (RAAS), endothelial ACE-2 is crucial for the regulation of cerebral blood flow in the cerebrovascular system. The RAAS is the main peptide hormone system responsible for blood pressure and volume regulation during circulation (31). Apart from ACE-2, the RAAS comprises several crucial constituents such as renin, angiotensinogen, angiotensin I (Ang I), angiotensin II (Ang II), ACE, angiotensin II type-1 receptor (AT1R), angiotensin II type-2 receptor (AT2R), and Mas receptor. The classical RAAS pathway involves the enzymatic action of renin on angiotensinogen resulting in the formation of Ang I. ACE enzymatically catalyzes the conversion of Angiotensin I to Angiotensin II, a peptide with significant biological activity that interacts with both AT1R and AT2R receptors. Overall, angiotensin II exhibited a greater affinity toward AT1R. The effect of angiotensin II on AT1R resulted in vasoconstriction and excretion of aldosterone from the adrenal glands (31, 32). Moreover, Angiotensin II has the potential to promote thrombosis through the augmentation of tissue factor expression and the release of fibrinogen activator inhibitor type 1 (33). In the alternative RAAS pathway, ACE-2 enzymatically cleaves Ang II to produce angiotensin 1–7 (Ang [1–7]). This substance then activates Mas receptors, leading to the mediation of vasodilatory, anti-inflammatory, and antioxidant effects (31, 34). In addition, ACE-2 possesses a pronounced antithrombotic action by diminishing platelet aggregation and enhancing nitric oxide release (35).

The interaction between SARS-CoV-2 and ACE-2 may result in a reduction of ACE-2, which in turn may lead to an elevation in ACE-dependent Ang II synthesis and a decrease in ACE-2-dependent Ang (1–7) synthesis (36). The imbalance induced by SARS-CoV-2 between the classical and alternative RAAS pathway eventually leads to ischemia by means of heightened cerebral vasoconstriction, hyperinflammation, and oxidative stress (37). The aforementioned impact, in conjunction with the damage inflicted on endothelial cells due to SARS-CoV-2 infection, has the potential to compromise the stability of atherosclerotic plaques that are already susceptible, thereby resulting in vascular occlusion (11).

### 2.4. COVID-19 associated cardiogenic embolism

SARS-CoV-2 infection has been found to potentially result in various cardiac complications, such as decompensated heart failure, myocarditis, acute myocardial infarction, and arrhythmias (38, 39). The cardiac symptoms associated with COVID-19 have the potential to result in subsequent strokes caused by cardioembolism. Aside from acute coronary events, cytokine storm-related systemic inflammatory responses or direct attack of SARS-CoV-2 on cardiomyocytes could lead to severely impaired cardiac function (40, 41). Moreover, respiratory failure and hypoxemia caused by SARS-CoV-2 infection have the potential to worsen cardiac impairment. The occurrence of thrombosis within the failing left ventricle poses a substantial risk for stroke, particularly in the context of a hypercoagulable condition (42). Research has demonstrated that SARS-CoV-2 infection is associated with an elevated likelihood of developing atrial fibrillation, a recognized risk factor for ischemic stroke. Systemic inflammation and myocardial dysfunction may be risk factors for atrial fibrillation in COVID-19 patients (43, 44). Malignant ventricular arrhythmias may be induced in cases of elevated cardiac output due to respiratory failure and hypoxemia, which in turn may cause cerebral ischemia and hypoxia (45, 46). Moreover, patients with severe COVID-19 frequently experience recurrent bacterial infections, thereby elevating their susceptibility to bacteremia and infective endocarditis, both of which heighten the likelihood of cardioembolism (47).

### 2.5. Other pathophysiological considerations of COVID-19-related stroke

Hemodynamic alterations following SARS-CoV-2 infection may be responsible for cerebral ischemia (48). For example, fever, diarrhea, or inadequate fluid intake due to malaise and anorexia can lead to dehydration. Patients with vascular risk factors (e.g., diabetes) may be particularly vulnerable because infections may exacerbate hyperglycemia, leading to additional dehydration. Dehydration may cause viscous and stagnant blood, which increases the risk of stroke (49). In addition, dehydrated patients may experience cerebral infarction caused by inadequate cerebral perfusion pressure, especially when hemodynamic fluctuations exceed their ability to self-regulate (50).

Discomfort and anorexia in COVID-19 patients may lead to the discontinuation of daily stroke prevention medications or the addition of medications such as decongestants and cough suppressants to treat these discomforts. Most of these medications contain vasoactive substances such as norepinephrine and other sympathomimetic drugs. However, the use of these drugs has been associated with unstable hypertension, ischemic stroke, and cerebral hemorrhage (51). Nonsteroidal anti-inflammatory drugs such as acetaminophen exhibit analgesic and antipyretic effects, but they can also cause vasoconstriction and increased blood pressure, which are implicated in an increased risk of stroke (52). Similarly, pseudoephedrine, a potent amine that relieves nasal congestion, has been shown to constrict vessels and raise blood pressure (53). In addition, some COVID-19 patients seek chiropractic manipulation therapy to relieve myalgia, which may potentially lead to carotid or vertebral artery dissection and stroke (54).

COVID-19 patients afflicted with severe pneumonia may necessitate intubation, mechanical ventilation, and extended hospitalization within the Intensive Care Unit (ICU). The critical illness-induced hypoxemia and systemic hypotension can exacerbate cerebral hypoxia-ischemia, leading to the onset of ischemic stroke, particularly in watershed areas, or presenting as cortical laminar necrosis (55). Furthermore, extended periods of hypoxemia and respiratory failure have been linked to the occurrence of cerebral microhemorrhages and white matter encephalopathy (56).

## 3. COVID-19-associated cerebrovascular complications

Acute cerebrovascular events are frequently being reported in patients with COVID-19 (57). The pathophysiologic mechanisms are not fully understood and may involve direct vascular injury mediated by viral invasion, hypercoagulation and cardiogenic embolism, as previously described. On the other hand, cerebral hemorrhage and cerebral venous thrombosis were less frequent in COVID-19 patients (57, 58). Cases with atypical neurovascular manifestations have also been reported, including carotid artery dissection, posterior reversible encephalopathy syndrome, and vasculitis (59–61).

### 3.1. Ischemic stroke

According to a comprehensive meta-analysis encompassing a sample size of over 60,000 individuals diagnosed with COVID-19, it was found that cerebrovascular events were detected in 1.3% of patients. Among these incidents, ischemic strokes were the most frequently occurring (57). Initially, different retrospective studies showed considerable variations in the prevalence of ischemic stroke among patients with COVID-19. Li et al. and Yaghi et al. reported varying incidences of ischemic stroke in COVID-19 inpatient cohorts. Li et al. found a 4.6% incidence in Wuhan, while Yaghi et al. reported a lower incidence of 0.9% in New York (30, 58). Cohort studies from Italy, France, Germany, Philadelphia, and other hospital systems in New York reported incidence rates within this range (62–65). So far, the largest cross-national studies and meta-analyses have estimated the risk in hospitalized COVID-19 patients to be between 0.5 and 1.3% (57, 66). The variability in incidence observed in various studies can be attributed to dissimilarities in the demographics of patients and their medical conditions. More COVID-19 stroke patients can be identified in regions with a high proportion of elderly people and sophisticated medical care. It may require more comprehensive and extensive multicenter studies to obtain more accurate incidence rates in the overall population. Although numerous reports have documented the prevalence of ischemic stroke in COVID-19 patients, these results are limited to hospitalized patients and do not reflect the actual incidence of ischemic stroke in all SARS-CoV-2 infected individuals. Statistics from ischemic stroke patients can be an underestimation because it is plausible that some patients suffering from mild stroke or transient ischemic attack did not seek hospital care due to lockdowns, hospital overload or concerns over infection (67).

Individuals with a prior history of ischemic stroke, diabetes, high D-dimer levels, and other conventional risk factors for stroke are seemingly at an increased susceptibility to ischemic stroke in the context of SARS-CoV-2 infection (68–70). In comparison to uninfected controls, patients diagnosed with COVID-19 exhibited a 3.6-fold augmented susceptibility to ischemic stroke (57), which is consistent with the findings of other meta-analyses (71). Furthermore, there seems to be a correlation between the severity of COVID-19 and the risk of stroke. According to the report, the incidence of stroke in patients with mild COVID-19 symptoms is estimated to be around 1%, whereas in patients who require ICU admission, the risk may increase up to 5.7% (71, 72). While viral infections are commonly linked to an increased risk of stroke, patients diagnosed with COVID-19 exhibit a notably heightened susceptibility to stroke. Research has indicated that individuals diagnosed with COVID-19 exhibit a stroke risk that is seven times greater than those diagnosed with influenza (73). The heightened susceptibility to stroke in COVID-19 patients may be due to factors such as excessive inflammation, hypercoagulable states, or more severe clinical symptoms.

In comparison to stroke patients without COVID-19, those with COVID-19 who also experienced stroke was observed to be younger (median in years: 63 versus 70, *p* = 0.001), had a higher proportion of male individuals, and exhibited more severe neurological deficits (median National Institutes of Health Stroke Scale score: 19 versus 8, *p* = 0.007) (30). The higher incidence of large vessel occlusion (LVO) in COVID-19 stroke patients and the systemic inflammation that exacerbates brain injury may be responsible for the more severe neurological deficits (98).

The prognosis of stroke patients is comparatively worse in the presence of COVID-19 as compared to those who do not have COVID-19. An earlier report from China described poorer outcomes and increased in-hospital mortality in COVID-19 stroke patients (104). Research conducted in the United States has demonstrated that a considerable proportion of patients, up to 63.6%, who contract COVID-19 and experience a stroke, pass away during their hospitalization (30). In their study, Qureshi et al. analyzed over 27,000 hospitalized patients and found that those who were diagnosed with COVID-19 and experienced an acute ischemic stroke exhibited a twofold elevation in the likelihood of being discharged to a non-home destination or death (106). The poor prognosis of COVID-19 stroke patients might be associated with more severe onset symptoms and lack of effective rehabilitation during the epidemic (101). Another explanation for the worsening stroke prognosis in COVID-19 patients is probably the diminished use of acute stroke therapies such as tissue fibrinogen activator (t-PA) and mechanical thrombectomy (MT). There may be a lower probability of administering t-PA and MT to stroke patients who have contracted COVID-19, particularly those who are severely ill (121). There are various potential factors that could contribute to this phenomenon, one of which may be a lag in the identification of stroke symptoms as a result of sedative administration. Furthermore, individuals with severe COVID-19 may be administered therapeutic anticoagulation to address systemic clotting or may have serious medical comorbidities that preclude them from receiving t-PA and MT (95). There were also other studies, however, that showed no difference in outcomes at 3 months in COVID-19 stroke patients compared to pre-COVID-19, despite the longer treatment time and higher hospital costs (79).

The neuroimaging features of COVID-19-related ischemic stroke are the presence of multiple thrombi, including multiregional involvement, atypical vascular involvement, and large vessel occlusion (78). Several case series have highlighted an increased prevalence of LVO strokes, mainly cryptogenic embolic strokes, in COVID-19 patients (71, 83, 102). The stroke features of 432 COVID-19 patients who were admitted to 71 centers in 17 countries were documented in an extensive multicenter research. Compared with pre-pandemic study findings, they observed higher rates of cerebral large vessel occlusion and lower rates of lacunar cerebral infarction (110). The aforementioned observation implies a plausible absence of correlation between COVID-19 and cerebral small vessel disease. Nonetheless, due to the relatively mild severity of lacunar stroke symptoms, it is possible that patients may not have been subjected to neuroimaging assessment via brain MRI, leading to an underestimation of the incidence of lacunar stroke among COVID-19 patients in the investigation. Therefore, COVID-19 patients with high risk factors for stroke should undergo brain MRI examination if possible to clarify the presence of lacunar stroke and to provide early targeted prevention and treatment.

### 3.2. Cerebral hemorrhage

Cerebral hemorrhage associated with COVID-19 is less common than cerebral infarction, and studies assessing the risk of hemorrhagic stroke (including parenchymal and subarachnoid hemorrhage) in patients with COVID-19 are relatively limited. According to studies, approximately 0.5% of COVID-19 inpatients suffer from cerebral hemorrhage (58). One study demonstrated no apparent increase in cerebral hemorrhage events in COVID-19 patients hospitalized in neurology as opposed to non-COVID-19 patients (76).

Hemorrhagic strokes may be induced by medications. Anticoagulation has been identified as a primary reason for intracranial hemorrhage in COVID-19 patients. Kvernland et al. demonstrated 89.5% of COVID-19 patients underwent anticoagulation prior to the occurrence of nontraumatic cerebral hemorrhage or spontaneous nonaneurysmal subarachnoid hemorrhage (95). In contrast to spontaneous cerebral hemorrhage, which predominantly presents as deep cerebral parenchymal hemorrhage, COVID-19 patients commonly exhibit lobar and multifocal hematomas in the absence of underlying vascular abnormalities (75, 95). Cerebral microhemorrhages have also been found in patients with severe COVID-19, with or without white matter encephalopathy. They are mostly found in the corpus callosum and paracortical regions, and are atypical locations for cerebral microhemorrhages (56, 94).

### 3.3. Cerebral venous thrombosis

A meta-analysis containing 67,845 COVID-19 patients revealed that approximately 0.03% of the patients developed CVT, which mainly involves the transverse and sigmoid sinuses (57). Several case reports have indicated that individuals, both adults and children, have developed CVT in association with COVID-19 (77, 86, 89, 90, 103). Headache was the main symptom, with varying degrees of focal neurological dysfunction and impaired consciousness. In general, those CVT patients exhibited varying degrees of elevation in acute-phase reactants (CRP and ferritin), hypercoagulable factors (D-dimer and APTT), and platelet counts, indicating a potential correlation with the hypercoagulable state that occurs during SARS-CoV-2 infection. When COVID-19 patients exhibit a persistent headache and altered consciousness with confusion or agitation, particularly with high D-dimer levels (>2.0 ng/mL), clinicians should consider the possibility of CVT for prompt diagnosis and anticoagulation therapy (111).

## 4. Acute stroke treatment during the COVID-19 pandemic

The therapeutic paradigm for acute ischemic stroke associated with COVID-19 is essentially the same as in the pre-COVID-19 era and includes inhibition of thrombosis (anticoagulation and antiplatelet therapy) and promotion of revascularization (thrombolysis and mechanical thrombectomy). Also, based on the current recognition of systemic inflammation, hypercoagulable states and altered RAAS pathways in COVID-19 patients, several therapeutic interventions targeting relevant targets have been tested.

### 4.1. Thromboprophylaxis

Antiplatelet aggregation is fundamental to the treatment of ischemic stroke, especially when anticoagulants cannot be administered owing to potential hemorrhagic transformation or other medical restrictions. In the case of platelet activation and aggregation induced by SARS-CoV-2 through inflammation and immune responses, the use of antiplatelet agents may be a preferred therapeutic strategy. Some studies suggest, however, that antiplatelet agents may not be suitable for COVID-19 patients with disseminated intravascular coagulation or severe thrombocytopenia due to the increased risk of bleeding (100).

A randomized clinical trial was conducted to assess the effectiveness of prophylactic anticoagulation in patients with non-severe COVID-19 who may develop ischemic stroke due to a hypercoagulable state. The study revealed that administering heparin anticoagulation resulted in a higher likelihood of surviving until hospital discharge and a decreased utilization of cardiovascular or respiratory organ support in comparison to conventional thromboprophylaxis treatment (97). Hence, it is advisable to contemplate prophylactic anticoagulation therapy for COVID-19 patients who do not necessitate ICU-level medical attention. Furthermore, it is recommended that stroke patients who exhibit a high likelihood of cardiogenic embolism or an embolism of uncertain origin, such as those presenting with multiple regional infarctions, significant ventricular motility disorders, atrial fibrillation, or a right-to-left shunt, undergo therapeutic anticoagulation treatment following the elimination of hemorrhage risk (112).

### 4.2. Thrombolysis

Revascularization is the core of early treatment for acute ischemic stroke (AIS) and can significantly improve prognosis. Intravenous thrombolysis with alteplase or Tenecteplase can be performed when a patient is excluded from hemorrhage by cranial CT within 4.5 h of the onset of stroke symptoms. In cases where proximal LVO is identified through CTA or MRA within 6 h of the onset of symptoms, it is recommended to proceed with intra-arterial mechanical thrombectomy subsequent to the administration of intravenous thrombolysis (116). However, the COVID-19 pandemic has significantly hampered recanalization therapy for patients with AIS.

A study from China reported that AIS patients’ pre-hospital (onset-to-visit) and post-hospital (door-to-puncture) delays were significantly longer and that a notably lower proportion of these patients received intravenous thrombolysis (87). Untimely stroke recognition due to isolation, delayed transport to the hospital, pre-admission COVID-19 screening, and the preparation of protective equipment for stroke team members may result in missing treatment time windows.

To date, there is a paucity of comprehensive studies examining the effectiveness and safety of thrombolytic medications in treating COVID-19-related AIS. Nevertheless, in a small series of four COVID-19 stroke patients, intravenous use of recombinant tissue-type plasminogen activator (rt-PA) was associated with catastrophic bleeding, suggesting that COVID-19 patients may have a greater susceptibility to hemorrhagic complications (108). In an observational cohort study evaluating the safety and outcomes of t-PA thrombolysis in 101 patients with COVID-19 AIS, COVID-19 patients had more severe symptoms compared with non-COVID-19 patients. However, after adjustment for confounding variables, there were no significant differences in hemorrhagic transformation and in-hospital mortality (109). According to Khedr et al.’s research, the findings were dissimilar. The research findings indicate that the COVID-19 group exhibited more severe neurological deficits compared to the non-COVID-19 group. Additionally, the COVID-19 patients displayed a higher susceptibility to hemorrhagic transformation. They considered that the high hemorrhagic transformation rate after intravenous thrombolytic therapy in COVID-19 patients may be related to the concomitant endothelial injury, large infarct size, high National Institutes of Health Stroke Scale (NIHSS) score, hyperglycemia on admission, and multiple other co-morbidities of COVID-19 (92). Despite the high incidence of hemorrhagic transformation, t-PA remains the cornerstone of treatment for AIS with or without COVID-19 infection, and therefore COVID-19 patients should be closely monitored after receiving intravenous thrombolysis.

### 4.3. Mechanical thrombectomy

LVO is common in COVID-19-related strokes, and studies have shown that it occurs in up to 60% of COVID-19 stroke patients (93). Among these patients, LVO mainly involves the anterior circulation, especially the middle cerebral artery and the internal carotid artery (93). LVO leads to widespread ischemia in the brain area, which is extremely prone to severe disability and mortality in the absence of further treatment. In recent years, a significant amount of high-quality data has demonstrated the efficacy of mechanical endovascular thrombectomy in enhancing clinical outcomes for patients with LVO (107). However, during the COVID-19 pandemic, many MT operations have been postponed or even canceled because of the need to screen for SARS-CoV-2 infection and relocate limited medical resources.

Kerlerouxd et al. reported an approximately 21% reduction in the number of MT cases in France in the first month after the COVID-19 pandemic (91). Kwan reported a 21% reduction in external stroke thrombectomy referrals in the United Kingdom (96). Qureshi et al. also reported a significant reduction in MT procedures in the United States (105). The most likely reason for the significant decrease in MT procedures is that stroke care centers strictly limit the number of patients eligible for MT, and patients beyond guideline indications are unlikely to receive MT. Other possible reasons are that patients (especially in cases of “mild” stroke symptoms) are reluctant to come to the hospital and emergency room due to the rigorous screening procedures or out of fear of exposure to the virus. Nevertheless, even during a pandemic, AIS patients with LAO must undergo emergency MT surgery without delay to reduce disability and mortality rates (118). This is because saving the ischemic brain tissue depends heavily on the timing of revascularization.

Several studies have reported prolonged door-to-penetration and door-to-reperfusion times during the COVID-19 pandemic (91, 114, 118). Several studies have also demonstrated that procedure optimization can decrease door-to-needle and door-to-puncture times during the COVID-19 pandemic, thereby improving patient prognosis (80, 119). One study demonstrated that treatment delays did not affect short-term outcomes (96). Although no significant differences were found in short-term outcomes (follow-up with the NIHSS on days 1–3 after MT), no long-term follow-up (modified Rankin scale, 90 days) was performed to assess long-term efficacy. One study evaluated the duration of MT operation in acute stroke patients with and without COVID-19. The results showed no statistically significant difference between the two groups (*p* = 0.45) (74). A study conducted found an elevated risk of vessel re-occlusion subsequent to MT treatment among COVID-19 patients with LVOs. This outcome could be partly ascribed to an overabundance of inflammation and hypercoagulation (85).

A meta-analysis incorporated one prospective cohort study and eight retrospective cohort studies, encompassing a cumulative sample size of 309 patients who encountered stroke with COVID-19. The thrombolysis in cerebral infarction (TICI) ≥2b rate in COVID-19 stroke patients treated with MT was 79% (95% CI: 73–85), the rate of symptomatic intracranial hemorrhage (sICH) was 6% (95% CI: 3–11), the rate of type 1 parenchymal hematoma was 11.1% (95% CI: 5–23), and the mortality was 29% (95% CI, 24–35). Comparing TICI ≥2b scores between COVID-19 and non-COVID-19 stroke patients treated with MT, they found no significant difference. However, mortality after MT surgery was substantially higher in COVID-19 stroke patients compared to non-COVID-19 stroke patients (84). The recanalization status, NIHSS score at 24 h after MT, and sICH were predictors of mortality after MT in patients with AIS (81). Despite prompt intravenous thrombolysis and favorable thrombectomy-reperfusion therapy, stroke patients with COVID-19 have worse clinical and imaging outcomes than those without COVID-19. It is probably mainly because of the susceptibility to re-occlusion in patients with COVID-19. Furthermore, delayed attendance with higher initial NIHSS scores and multisystem comorbidities of COVID-19 also contribute to increased stroke severity and mortality in these patients. Increased body temperature may also be associated with a worse prognosis in COVID-19 stroke patients after MT (82).

Overall, MT therapy remains the primary option for COVID-19 stroke patients who develop LAO, with reperfusion rates comparable to those of non-COVID-19 patients. It is effective in saving brain tissue in the ischemic region and improving prognosis.

### 4.4. Other therapeutic options for COVID-19-related stroke

Given the detrimental effect of systemic inflammation in stroke development of COVID-19 patients, anti-inflammatory therapy is thought to work synergistically with antithrombotic and revascularization therapies to improve neurological deficits. Randomized controlled trials have shown that anti-inflammatory drugs, including corticosteroids, reduce mortality in COVID-19 patients with serious complications such as respiratory distress syndrome and cardiac failure (88, 99). Systemic corticosteroids or other anti-inflammatory drugs may be also effective in stroke patients with cerebral vasculitis. Given that inflammatory factors such as IL-6, TNF-α, and IL-1β are significantly elevated in severe COVID-19 patients (16), treatments such as IL-6R monoclonal antibodies (Tocilizumab and Sarilumab), anti-TNF-α monoclonal antibody (Adalimumab and Infliximab) and IL-1 receptor antagonists (Anakinra and Canakinumab) were proposed to be potentially beneficial for COVID-19 stroke patients (120).

Considering the consumption of ACE-2 by SARS-CoV-2 may disrupt vascular endothelial function and lead to ischemic stroke. Another rational treatment for COVID-19 patients is nafamostat, which prevents COVID-19 from binding to the ACE-2 receptor, thereby reducing host cell infection and decreasing endogenous ACE-2 depletion (117). In addition, studies have shown that activation of AT2R has antithrombotic effects and helps to diminish the procoagulant state in patients with COVID-19 (113). Compound 21, a selective angiotensin II receptor agonist, may be beneficial in patients suffering from COVID-19 with ischemic stroke (115).

## 5. Conclusion

Acute cerebrovascular events, including ischemic and hemorrhagic stroke and cerebral venous thrombosis, are more frequency occur in patients with COVID-19. The underlying mechanisms of these events are still not fully understood. Nevertheless, they may include a hypercoagulable state, hyperinflammation and cytokine storm, endothelial damage, cardioembolism from pre-existing or new-onset arrhythmias and RAAS abnormalities due to SARA-CoV-2 binding to endothelial ACE-2. These abnormalities ultimately lead to impaired vasoconstriction, blood–brain barrier disruption, platelet activation and thrombosis. Equally, widespread viruses are linked to an increased risk of ischemic stroke. The pathophysiological concepts regarding SARA-CoV-2 and stroke can help our comprehension of the association between other acute viral infections and stroke.

In the context of COVID-19 infection, stroke is more likely to occur in cases of critical illness and those with a history of diabetes, hypertension, atrial fibrillation, cerebrovascular disease, or coronary artery disease. Furthermore, compared to non-COVID-19 stroke patients, COVID-19 stroke patients have more severe symptoms of neurological deficit, higher incidence of LVO, and worse prognosis.

The treatment of COVID-19-related strokes may be challenging. COVID-19 patients have a higher risk of hemorrhage when receiving intravenous thrombolytic therapy in the acute phase of AIS. The recanalization rate of MT in stroke patients with and without COVID-19 was not significantly different, but mortality was higher in patients with COVID-19. Nevertheless, intravenous thrombolysis and mechanical thrombectomy remain the cornerstones of the acute treatment of patients with COVID-19 AIS. The use of anticoagulants for stroke prevention in COVID-19 patients is still debatable. More research is required to identify the appropriate drug at the appropriate time and duration after infection.

## Author contributions

YaL contributed to the conception and design of the study. WZ wrote the first draft of the manuscript. LL, JL, and YuL wrote sections of the manuscript. All authors contributed to the manuscript revision, read, and approved the submitted version.

## Funding

This work was supported by grants from the Sanming Project of Medicine in Shenzhen (SZSM201812047), Shenzhen Key Medical Discipline Construction Fund (SZXK074) and the Science and Technology Project of Shenzhen (JCYJ20190808123611319).

## Conflict of interest

The authors declare that the research was conducted in the absence of any commercial or financial relationships that could be construed as a potential conflict of interest.

## Publisher’s note

All claims expressed in this article are solely those of the authors and do not necessarily represent those of their affiliated organizations, or those of the publisher, the editors and the reviewers. Any product that may be evaluated in this article, or claim that may be made by its manufacturer, is not guaranteed or endorsed by the publisher.

## References

[1] World Health Organization. (2023). Who coronavirus (COVID-19) dashboard [online]. Available at: https://covid19.who.int/ (Accessed June 12, 2023)

[2] Rofail D Mcgale N Podolanczuk AJ Rams A Przydzial K Sivapalasingam S. Patient experience of symptoms and impacts of COVID-19: a qualitative investigation with symptomatic outpatients. BMJ Open. (2022) 12:E055989. doi: 10.1136/bmjopen-2021-055989 PMC9062460 35501077

[3] Vallamkondu J John A Wani WY Ramadevi SP Jella KK Reddy PH. SARS-CoV-2 pathophysiology and assessment of coronaviruses in CNS diseases with a focus on therapeutic targets. Biochimica Et Biophysica Acta Mol Basis Dis. (2020) 1866:165889. doi: 10.1016/j.bbadis.2020.165889 PMC7320676 32603829

[4] Karuppan MKM Devadoss D Nair M Chand HS Lakshmana MK. SARS-CoV-2 infection in the central and peripheral nervous system-associated morbidities and their potential mechanism. Mol Neurobiol. (2021) 58:2465–80. doi: 10.1007/s12035-020-02245-1, PMID: 33439437 PMC7805264

[5] Luo W Liu X Bao K Huang C. Ischemic stroke associated with COVID-19: a systematic review and meta-analysis. J Neurol. (2022) 269:1731–40. doi: 10.1007/s00415-021-10837-7, PMID: 34652503 PMC8517946

[6] Conway EM Mackman N Warren RQ Wolberg AS Mosnier LO Campbell RA. Understanding COVID-19-associated coagulopathy. Nat Rev Immunol. (2022) 22:639–49. doi: 10.1038/s41577-022-00762-9, PMID: 35931818 PMC9362465

[7] Zhang C Wu Z Li J-W Zhao H Wang G-Q. Cytokine release syndrome in severe COVID-19: Interleukin-6 receptor antagonist Tocilizumab may be the key to reduce mortality. Int J Antimicrob Agents. (2020) 55:105954. doi: 10.1016/j.ijantimicag.2020.105954, PMID: 32234467 PMC7118634

[8] Biancolella M Colona VL Mehrian-Shai R Watt JL Luzzatto L Novelli G. COVID-19 2022 update: transition of the pandemic to the endemic phase. Hum Genomics. (2022) 16:19. doi: 10.1186/s40246-022-00392-1 35650595 PMC9156835

[9] Ziegler CGK Allon SJ Nyquist SK Mbano IM Miao VN Tzouanas CN. SARS-CoV-2 receptor Ace2 is an interferon-stimulated gene in human airway epithelial cells and is detected in specific cell subsets across tissues. Cells. (2020) 181:35. doi: 10.1016/j.cell.2020.04.035, PMID: 32413319 PMC7252096

[10] Lukiw WJ Pogue A Hill JM. SARS-CoV-2 infectivity and neurological targets in the brain. Cell Mol Neurobiol. (2022) 42:217–24. doi: 10.1007/s10571-020-00947-7, PMID: 32840758 PMC7445393

[11] Varga Z Flammer AJ Steiger P Haberecker M Andermatt R Zinkernagel AS. Endothelial cell infection and Endotheliitis in COVID-19. Lancet (London, England). (2020) 395:1417–8. doi: 10.1016/S0140-6736(20)30937-5 32325026 PMC7172722

[12] Pezzini A Padovani A. Lifting the mask on neurological manifestations of COVID-19. Nat Rev Neurol. (2020) 16:636–44. doi: 10.1038/s41582-020-0398-3, PMID: 32839585 PMC7444680

[13] Bodro M Compta Y Sánchez-Valle R. Presentations and mechanisms of CNS disorders related to COVID-19. Neurol Neuroimmunol Neuroinflam. (2021) 8:e923. doi: 10.1212/NXI.0000000000000923 PMC7808129 33310765

[14] Chua AMU Jamora RDG Jose ACE Anlacan VMM. Cerebral vasculitis in a COVID-19 confirmed postpartum patient: a case report. Case Reports Neurol. (2021) 13:324–8. doi: 10.1159/000515815, PMID: 34248564 PMC8255701

[15] Alam SB Willows S Kulka M Sandhu JK. Severe acute respiratory syndrome coronavirus 2 may be an underappreciated pathogen of the central nervous system. Eur J Neurol. (2020) 27:2348–60. doi: 10.1111/ene.14442, PMID: 32668062 PMC7405269

[16] Huang C Wang Y Li X Ren L Zhao J Hu Y. Clinical features of patients infected with 2019 novel coronavirus in Wuhan, China. Lancet (London, England). (2020) 395:497–506. doi: 10.1016/S0140-6736(20)30183-5 31986264 PMC7159299

[17] Galea I. The blood-brain barrier in systemic infection and inflammation. Cell Mol Immunol. (2021) 18:2489–501. doi: 10.1038/s41423-021-00757-x, PMID: 34594000 PMC8481764

[18] Ntaios G Michel P Georgiopoulos G Guo Y Li W Xiong J. Characteristics and outcomes in patients with COVID-19 and acute ischemic stroke: the global COVID-19 stroke registry. Stroke. (2020) 51:E254–8. doi: 10.1161/STROKEAHA.120.031208 32787707 PMC7359900

[19] Murta V Villarreal A Ramos AJ. Severe acute respiratory syndrome coronavirus 2 impact on the central nervous system: are astrocytes and microglia Main players or merely bystanders? ASN Neuro. (2020) 12:1759091420954960. doi: 10.1177/1759091420954960, PMID: 32878468 PMC7476346

[20] Helms J Kremer S Merdji H Clere-Jehl R Schenck M Kummerlen C. Neurologic features in severe SARS-CoV-2 infection. N Engl J Med. (2020) 382:2268–70. doi: 10.1056/NEJMc2008597, PMID: 32294339 PMC7179967

[21] Becker RC. COVID-19 update: COVID-19-associated coagulopathy. J Thromb Thrombolysis. (2020) 50:54–67. doi: 10.1007/s11239-020-02134-3, PMID: 32415579 PMC7225095

[22] Klok FA Kruip MJHA Van Der Meer NJM Arbous MS Gommers DAMPJ Kant KM. Incidence of thrombotic complications in critically ill ICU patients with COVID-19. Thromb Res. (2020) 191:145–7. doi: 10.1016/j.thromres.2020.04.013, PMID: 32291094 PMC7146714

[23] Zhang H Penninger JM Li Y Zhong N Slutsky AS. Angiotensin-converting enzyme 2 (Ace2) as a SARS-CoV-2 receptor: molecular mechanisms and potential therapeutic target. Intensive Care Med. (2020) 46:586–90. doi: 10.1007/s00134-020-05985-9, PMID: 32125455 PMC7079879

[24] Singhania N Bansal S Nimmatoori DP Ejaz AA Mccullough PA Singhania G. Current overview on hypercoagulability in COVID-19. Am J Cardiovasc Drugs. (2020) 20:393–403. doi: 10.1007/s40256-020-00431-z, PMID: 32748336 PMC7398761

[25] Hong L-Z Shou Z-X Zheng D-M Jin X. The most important biomarker associated with coagulation and inflammation among COVID-19 patients. Mol Cell Biochem. (2021) 476:2877–85. doi: 10.1007/s11010-021-04122-4, PMID: 33742367 PMC7978444

[26] Violi F Pastori D Cangemi R Pignatelli P Loffredo L. Hypercoagulation and antithrombotic treatment in coronavirus 2019: A new challenge. Thromb Haemost. (2020) 120:949–56. doi: 10.1055/s-0040-1710317, PMID: 32349133 PMC7295290

[27] Wells PS Anderson DR Rodger M Forgie M Kearon C Dreyer J. Evaluation of D-dimer in the diagnosis of suspected deep-vein thrombosis. N Engl J Med. (2003) 349:1227–35. doi: 10.1056/NEJMoa023153, PMID: 14507948

[28] Goldberg MF Goldberg MF Cerejo R Tayal AH. Cerebrovascular disease in COVID-19. AJNR Am J Neuroradiol. (2020) 41:1170–2. doi: 10.3174/ajnr.A6588, PMID: 32409316 PMC7357639

[29] Zhou F Yu T Du R Fan G Liu Y Liu Z. Clinical course and risk factors for mortality of adult inpatients with COVID-19 in Wuhan, China: a retrospective cohort study. Lancet (London, England). (2020) 395:1054–62. doi: 10.1016/S0140-6736(20)30566-3 32171076 PMC7270627

[30] Yaghi S Ishida K Torres J Mac Grory B Raz E Humbert K. SARS-CoV-2 and stroke in a New York healthcare system. Stroke. (2020) 51:2002–11. doi: 10.1161/STROKEAHA.120.030335, PMID: 32432996 PMC7258764

[31] Gheblawi M Wang K Viveiros A Nguyen Q Zhong J-C Turner AJ. Angiotensin-converting enzyme 2: SARS-CoV-2 receptor and regulator of the renin-angiotensin system: celebrating the 20th anniversary of the discovery of Ace2. Circ Res. (2020) 126:1456–74. doi: 10.1161/CIRCRESAHA.120.317015, PMID: 32264791 PMC7188049

[32] Schalekamp MADH Danser AHJ. How does the angiotensin ii type 1 receptor 'Trump' the type 2 receptor in blood pressure control? J Hypertens. (2013) 31:705–12. doi: 10.1097/HJH.0b013e32835d6d11, PMID: 23325393

[33] Kucharewicz I Pawlak R Matys T Pawlak D Buczko W. Antithrombotic effect of captopril and losartan is mediated by angiotensin-(1-7). Hypertension. (2002) 40:774–9. doi: 10.1161/01.HYP.0000035396.27909.40 12411476

[34] Sanchis-Gomar F Lavie CJ Perez-Quilis C Henry BM Lippi G. Angiotensin-converting enzyme 2 and Antihypertensives (angiotensin receptor blockers and angiotensin-converting enzyme inhibitors) in coronavirus disease 2019. Mayo Clin Proc. (2020) 95:1222–30. doi: 10.1016/j.mayocp.2020.03.026, PMID: 32376099 PMC7129862

[35] Fraga-Silva RA Pinheiro SVB Gonçalves ACC Alenina N Bader M Santos RAS. The antithrombotic effect of angiotensin-(1-7) involves mas-mediated no release from platelets. Mol Med. (2008) 14:28–35. doi: 10.2119/2007-00073 18026570 PMC2078558

[36] Zhang Y Xiao M Zhang S Xia P Cao W Jiang W. Coagulopathy and Antiphospholipid antibodies in patients with COVID-19. N Engl J Med. (2020) 382:E38. doi: 10.1056/NEJMc2007575 32268022 PMC7161262

[37] Divani AA Andalib S Di Napoli M Lattanzi S Hussain MS Biller J. Coronavirus disease 2019 and stroke: clinical manifestations and pathophysiological insights. J Stroke Cerebrovasc Dis. (2020) 29:104941. doi: 10.1016/j.jstrokecerebrovasdis.2020.104941, PMID: 32689643 PMC7214348

[38] Kochi AN Tagliari AP Forleo GB Fassini GM Tondo C. Cardiac and arrhythmic complications in patients with COVID-19. J Cardiovasc Electrophysiol. (2020) 31:1003–8. doi: 10.1111/jce.14479, PMID: 32270559 PMC7262150

[39] Patone M Mei XW Handunnetthi L Dixon S Zaccardi F Shankar-Hari M. Risks of myocarditis, pericarditis, and cardiac arrhythmias associated with COVID-19 vaccination or SARS-CoV-2 infection. Nat Med. (2022) 28:410–22. doi: 10.1038/s41591-021-01630-0, PMID: 34907393 PMC8863574

[40] Castiello T Georgiopoulos G Finocchiaro G Claudia M Gianatti A Delialis D. COVID-19 and myocarditis: a systematic review and overview of current challenges. Heart Fail Rev. (2022) 27:251–61. doi: 10.1007/s10741-021-10087-9, PMID: 33761041 PMC7988375

[41] Zeng J-H Liu Y-X Yuan J Wang F-X Wu W-B Li J-X. First case of COVID-19 complicated with fulminant myocarditis: a case report and insights. Infection. (2020) 48:773–7. doi: 10.1007/s15010-020-01424-5, PMID: 32277408 PMC7146072

[42] Merkler AE Diaz I Wu X Murthy SB Gialdini G Navi BB. Duration of heightened ischemic stroke risk after acute myocardial infarction. J Am Heart Assoc. (2018) 7:E010782. doi: 10.1161/JAHA.118.010782 30571491 PMC6404432

[43] Denegri A Morelli M Pezzuto G Malavasi VL Boriani G. Atrial fibrillation is related to higher mortality in COVID-19/SARS-CoV-2 pneumonia infection. Cardiol J. (2021) 28:973–5. doi: 10.5603/CJ.a2021.0102, PMID: 34523114 PMC8747817

[44] García-Granja PE Veras C Aparisi Á Amat-Santos IJ Catalá P Marcos M. Atrial fibrillation in patients with SARS-CoV-2 infection. Med Clin. (2021) 157:58–63. doi: 10.1016/j.medcli.2021.01.003, PMID: 33637334 PMC7843022

[45] Guo T Fan Y Chen M Wu X Zhang L He T. Cardiovascular implications of fatal outcomes of patients with coronavirus disease 2019 (COVID-19). JAMA Cardiol. (2020) 5:811–8. doi: 10.1001/jamacardio.2020.1017, PMID: 32219356 PMC7101506

[46] Lazzerini PE Boutjdir M Capecchi PL. COVID-19, arrhythmic risk, and inflammation: mind the gap! Circulation. (2020) 142:7–9. doi: 10.1161/CIRCULATIONAHA.120.047293, PMID: 32286863

[47] Amir M Djaharuddin I Sudharsono A Ramadany S. COVID-19 concomitant with infective endocarditis: A case report and review of management. Int J Infect Dis. (2020) 98:109–12. doi: 10.1016/j.ijid.2020.06.061, PMID: 32574691 PMC7305871

[48] Sastry S Cuomo F Muthusamy J. COVID-19 and thrombosis: the role of hemodynamics. Thromb Res. (2022) 212:51–7. doi: 10.1016/j.thromres.2022.02.016, PMID: 35219932 PMC8864963

[49] Lip GYH Blann AD Farooqi IS Zarifis J Sagar G Beevers DG. Sequential alterations in Haemorheology, endothelial dysfunction, platelet activation and Thrombogenesis in relation to prognosis following acute stroke: the West Birmingham stroke project. Blood Coagulat Fibrinol. (2002) 13:339–47. doi: 10.1097/00001721-200206000-00010, PMID: 12032400

[50] Trangmar SJ Chiesa ST Llodio I Garcia B Kalsi KK Secher NH. Dehydration accelerates reductions in cerebral blood flow during prolonged exercise in the heat without compromising brain metabolism. Am J Physiol Heart Circulat Physiol. (2015) 309:H1598–607. doi: 10.1152/ajpheart.00525.2015, PMID: 26371170 PMC4670459

[51] Ducros A. Reversible cerebral vasoconstriction syndrome. Lancet Neurol. (2012) 11:906–17. doi: 10.1016/S1474-4422(12)70135-7, PMID: 22995694

[52] Spence JD Grosser T Fitzgerald GA. Acetaminophen, nonsteroidal anti-inflammatory drugs, and hypertension. Hypertension. (2022) 79:1922–6. doi: 10.1161/HYPERTENSIONAHA.122.19315 35862146

[53] Laccourreye O Werner A Giroud JP Couloigner V Bonfils P Bondon-Guitton E. Benefits, limits and danger of ephedrine and pseudoephedrine as nasal decongestants. Eur Ann Otorhinolaryngol Head Neck Dis. (2015) 132:31–4. doi: 10.1016/j.anorl.2014.11.001, PMID: 25532441

[54] Chen Y Mofatteh M Nguyen TN Wellington J Wei W Liang W. Carotid artery dissection and ischemic stroke following cervical chiropractic manipulation: two case reports. Vasc Endovasc Surg. (2022) 56:303–7. doi: 10.1177/15385744211072660, PMID: 34971321

[55] Fan S Xiao M Han F Xia P Bai X Chen H. Neurological manifestations in critically ill patients with COVID-19: a retrospective study. Front Neurol. (2020) 11:806. doi: 10.3389/fneur.2020.00806 32754114 PMC7365850

[56] Agarwal S Jain R Dogra S Krieger P Lewis A Nguyen V. Cerebral microbleeds and Leukoencephalopathy in critically ill patients with COVID-19. Stroke. (2020) 51:2649–55. doi: 10.1161/STROKEAHA.120.030940, PMID: 32755456 PMC7434006

[57] Katsanos AH Palaiodimou L Zand R Yaghi S Kamel H Navi BB. The impact of SARS-CoV-2 on stroke epidemiology and care: a meta-analysis. Ann Neurol. (2021) 89:380–8. doi: 10.1002/ana.25967, PMID: 33219563 PMC7753413

[58] Li Y Li M Wang M Zhou Y Chang J Xian Y. Acute cerebrovascular disease following COVID-19: A single center, retrospective, observational study. Stroke Vascular Neurol. (2020) 5:279–84. doi: 10.1136/svn-2020-000431, PMID: 32616524 PMC7371480

[59] Asan L Deuschl C Forsting M Kleinschnitz C Köhrmann M. Oropharyngeal swab for SARS-CoV-2 test causing atypical internal carotid artery dissection and stroke in a patient after mild COVID-19. Ther Adv Neurol Disord. (2021) 14:17562864211033521. doi: 10.1177/17562864211033521, PMID: 35173807 PMC8842151

[60] Gulko E Overby P Ali S Mehta H Al-Mufti F Gomes W. Vessel wall enhancement and focal cerebral arteriopathy in a pediatric patient with acute infarct and COVID-19 infection. AJNR Am J Neuroradiol. (2020) 41:2348–50. doi: 10.3174/ajnr.A6778, PMID: 32816770 PMC7963235

[61] Harapan BN Yoo HJ. Neurological symptoms, manifestations, and complications associated with severe acute respiratory syndrome coronavirus 2 (SARS-CoV-2) and coronavirus disease 19 (COVID-19). J Neurol. (2021) 268:3059–71. doi: 10.1007/s00415-021-10406-y, PMID: 33486564 PMC7826147

[62] Helms J Tacquard C Severac F Leonard-Lorant I Ohana M Delabranche X. High risk of thrombosis in patients with severe SARS-CoV-2 infection: a multicenter prospective cohort study. Intensive Care Med. (2020) 46:1089–98. doi: 10.1007/s00134-020-06062-x, PMID: 32367170 PMC7197634

[63] Lodigiani C Iapichino G Carenzo L Cecconi M Ferrazzi P Sebastian T. Venous and arterial thromboembolic complications in COVID-19 patients admitted to an academic hospital in Milan, Italy. Thromb Res. (2020) 191:9–14. doi: 10.1016/j.thromres.2020.04.024 32353746 PMC7177070

[64] Rothstein A Oldridge O Schwennesen H Do D Cucchiara BL. Acute cerebrovascular events in hospitalized COVID-19 patients. Stroke. (2020) 51:E219–22. doi: 10.1161/STROKEAHA.120.030995 32684145 PMC7386677

[65] Siepmann T Sedghi A Simon E Winzer S Barlinn J De With K. Increased risk of acute stroke among patients with severe COVID-19: a multicenter study and meta-analysis. Eur J Neurol. (2021) 28:238–47. doi: 10.1111/ene.14535, PMID: 32920964

[66] Nogueira RG Abdalkader M Qureshi MM Frankel MR Mansour OY Yamagami H. Global impact of COVID-19 on stroke care. Int J Stroke. (2021) 16:573–84. doi: 10.1177/1747493021991652, PMID: 33459583 PMC8010375

[67] Diegoli H Magalhães PSC Martins SCO Moro CHC França PHC Safanelli J. Decrease in hospital admissions for transient ischemic attack, mild, and moderate stroke during the COVID-19 era. Stroke. (2020) 51:2315–21. doi: 10.1161/STROKEAHA.120.030481, PMID: 32530738 PMC7302100

[68] Annie F Bates MC Nanjundappa A Bhatt DL Alkhouli M. Prevalence and outcomes of acute ischemic stroke among patients ≤50 years of age with laboratory confirmed COVID-19 infection. Am J Cardiol. (2020) 130:169–70. doi: 10.1016/j.amjcard.2020.06.010, PMID: 32690214 PMC7293843

[69] Kim Y Khose S Abdelkhaleq R Salazar-Marioni S Zhang G-Q Sheth SA. Predicting in-hospital mortality using D-dimer in COVID-19 patients with acute ischemic stroke. Front Neurol. (2021) 12:702927. doi: 10.3389/fneur.2021.702927, PMID: 34335456 PMC8322655

[70] Shen J Hou Y Zhou Y Mehra R Jehi L Cheng F. The epidemiological and mechanistic understanding of the neurological manifestations of COVID-19: A comprehensive meta-analysis and A network medicine observation. Front Neurosci. (2021) 15:606926. doi: 10.3389/fnins.2021.606926, PMID: 33732102 PMC7959722

[71] Nannoni S De Groot R Bell S Markus HS. Stroke in COVID-19: a systematic review and meta-analysis. Int J Stroke. (2021) 16:137–49. doi: 10.1177/1747493020972922, PMID: 33103610 PMC7859578

[72] Mao L Jin H Wang M Hu Y Chen S He Q. Neurologic manifestations of hospitalized patients with coronavirus disease 2019 in Wuhan, China. JAMA Neurol. (2020) 77:683–90. doi: 10.1001/jamaneurol.2020.1127, PMID: 32275288 PMC7149362

[73] Merkler AE Parikh NS Mir S Gupta A Kamel H Lin E. Risk of ischemic stroke in patients with coronavirus disease 2019 (COVID-19) vs patients with influenza. JAMA Neurol. (2020) 77:1–7. doi: 10.1001/jamaneurol.2020.2730, PMID: 32614385 PMC7333175

[74] Al Kasab S Almallouhi E Alawieh A Levitt MR Jabbour P Sweid A. International experience of mechanical thrombectomy during the COVID-19 pandemic: insights from star and Enrg. J Neurointervent Surg. (2020) 12:1039–44. doi: 10.1136/neurintsurg-2020-016671, PMID: 32843359 PMC7453763

[75] Benger M Williams O Siddiqui J Sztriha L. Intracerebral Haemorrhage and COVID-19: clinical characteristics from A case series. Brain Behav Immun. (2020) 88:940–4. doi: 10.1016/j.bbi.2020.06.005, PMID: 32525049 PMC7276127

[76] Benussi A Pilotto A Premi E Libri I Giunta M Agosti C. Clinical characteristics and outcomes of inpatients with neurologic disease and COVID-19 in Brescia, Lombardy, Italy. Neurology. (2020) 95:E910–20. doi: 10.1212/WNL.0000000000009848 32444493

[77] Cavalcanti DD Raz E Shapiro M Dehkharghani S Yaghi S Lillemoe K. Cerebral venous thrombosis associated with COVID-19. AJNR Am J Neuroradiol. (2020) 41:1370–6. doi: 10.3174/ajnr.A6644, PMID: 32554424 PMC7658892

[78] Chang S Schecht M Jain R Belani P. Acute neurological complications of coronavirus disease. Neuroimaging Clin N Am. (2023) 33:57–68. doi: 10.1016/j.nic.2022.07.003, PMID: 36404047 PMC9288970

[79] Chen Y Nguyen TN Mofatteh M Abdalkader M Wellington J Yan Z. Association of early increase in body temperature with symptomatic intracranial hemorrhage and unfavorable outcome following endovascular therapy in patients with large vessel occlusion stroke. J Integr Neurosci. (2022) 21:156. doi: 10.31083/j.jin2106156, PMID: 36424759

[80] Chen Y Nguyen TN Siegler JE Mofatteh M Wellington J Yang R. The impact of COVID-19 pandemic on ischemic stroke patients in A comprehensive hospital. Risk Manag Healthcare Policy. (2022) 15:1741–9. doi: 10.2147/RMHP.S380691 PMC9482438 36124298

[81] Chen Y Nguyen TN Wellington J Mofatteh M Yao W Hu Z. Shortening door-to-needle time by multidisciplinary collaboration and workflow optimization during the COVID-19 pandemic. J Stroke Cerebrovasc Dis. (2022) 31:106179. doi: 10.1016/j.jstrokecerebrovasdis.2021.106179, PMID: 34735901 PMC8526426

[82] Chen Y Zhou S Yang S Mofatteh M Hu Y Wei H. Developing and predicting of early mortality after endovascular Thrombectomy in patients with acute ischemic stroke. Front Neurosci. (2022) 16:1034472. doi: 10.3389/fnins.2022.1034472 36605548 PMC9810273

[83] Dhamoon MS Thaler A Gururangan K Kohli A Sisniega D Wheelwright D. Acute cerebrovascular events with COVID-19 infection. Stroke. (2021) 52:48–56. doi: 10.1161/STROKEAHA.120.031668, PMID: 33280551

[84] El-Qushayri AE Reda A Dahy A Azzam AY Ghozy S. The impact of COVID 19 on the outcomes of Thrombectomy in stroke patients: a systematic review and Meta-analysis. Rev Med Virol. (2023) 33:E2379. doi: 10.1002/rmv.2379 35833712 PMC9349746

[85] Escalard S Maïer B Redjem H Delvoye F Hébert S Smajda S. Treatment of acute ischemic stroke due to large vessel occlusion with COVID-19: experience from Paris. Stroke. (2020) 51:2540–3. doi: 10.1161/STROKEAHA.120.030574, PMID: 32466736 PMC7282400

[86] Garaci F Di Giuliano F Picchi E Da Ros V Floris R. Venous cerebral thrombosis in COVID-19 patient. J Neurol Sci. (2020) 414:116871. doi: 10.1016/j.jns.2020.116871, PMID: 32422428 PMC7194045

[87] Gu S Dai Z Shen H Bai Y Zhang X Liu X. Delayed stroke treatment during COVID-19 pandemic in China. Cerebrovas Dis. (2021) 50:715–21. doi: 10.1159/000517075, PMID: 34247153 PMC8339026

[88] Horby P Lim WS Emberson JR Mafham M Bell JL Linsell L. Dexamethasone in hospitalized patients with COVID-19. N Engl J Med. (2021) 384:693–704. doi: 10.1056/NEJMoa2021436, PMID: 32678530 PMC7383595

[89] Hughes C Nichols T Pike M Subbe C Elghenzai S. Cerebral venous sinus thrombosis as A presentation of COVID-19. Europ J Case Reports Internal Med. (2020) 7:001691. doi: 10.12890/2020_001691, PMID: 32399457 PMC7213833

[90] Ippolito Bastidas H Márquez-Pérez T García-Salido A Luglietto D García Moreno R Martínez De Azagra-Garde A. Cerebral venous sinus thrombosis in a pediatric patient with COVID-19. Neurol Clin Pract. (2021) 11:E208–10. doi: 10.1212/CPJ.0000000000000899 33842096 PMC8032432

[91] Kerleroux B Fabacher T Bricout N Moïse M Testud B Vingadassalom S. Mechanical Thrombectomy for acute ischemic stroke amid the COVID-19 outbreak: decreased activity, and increased care delays. Stroke. (2020) 51:2012–7. doi: 10.1161/STROKEAHA.120.030373, PMID: 32432994

[92] Khedr EM Abdelwarith A Moussa G Saber M. Recombinant tissue plasminogen activator (Rtpa) management for first onset acute ischemic stroke with COVID-19 and non-COVID-19 patients. J Stroke Cerebrovasc Dis. (2023) 32:107031. doi: 10.1016/j.jstrokecerebrovasdis.2023.107031, PMID: 36701854 PMC9868389

[93] Kihira S Schefflein J Mahmoudi K Rigney B Delman N Mocco J. Association of coronavirus disease (COVID-19) with large vessel occlusion strokes: a case-control study. AJR Am J Roentgenol. (2021) 216:150–6. doi: 10.2214/AJR.20.23847, PMID: 32755225

[94] Kremer S Lersy F De Sèze J Ferré J-C Maamar A Carsin-Nicol B. Brain MRI findings in severe COVID-19: a retrospective observational study. Radiology. (2020) 297:E242–51. doi: 10.1148/radiol.2020202222, PMID: 32544034 PMC7301613

[95] Kvernland A Kumar A Yaghi S Raz E Frontera J Lewis A. Anticoagulation use and hemorrhagic stroke in SARS-CoV-2 patients treated at a New York healthcare system. Neurocrit Care. (2021) 34:748–59. doi: 10.1007/s12028-020-01077-0, PMID: 32839867 PMC7444897

[96] Kwan J Brown M Bentley P Brown Z D'anna L Hall C. Impact of COVID-19 pandemic on A regional stroke Thrombectomy service in the United Kingdom. Cerebrovasc Dis. (2021) 50:178–84. doi: 10.1159/000512603, PMID: 33311017 PMC7801959

[97] Lawler PR Goligher EC Berger JS Neal MD Mcverry BJ Nicolau JC. Therapeutic anticoagulation with heparin in noncritically ill patients with COVID-19. N Engl J Med. (2021) 385:790–802. doi: 10.1056/NEJMoa2105911, PMID: 34351721 PMC8362594

[98] Majidi S Fifi JT Ladner TR Lara-Reyna J Yaeger KA Yim B. Emergent large vessel occlusion stroke during New York City's COVID-19 outbreak: clinical characteristics and Paraclinical findings. Stroke. (2020) 51:2656–63. doi: 10.1161/STROKEAHA.120.030397, PMID: 32755349 PMC7434004

[99] Marconi VC Ramanan AV De Bono S Kartman CE Krishnan V Liao R. Efficacy and safety of Baricitinib for the treatment of hospitalised adults with COVID-19 (Cov-barrier): a randomised, double-blind, parallel-group, placebo-controlled phase 3 trial. Lancet Respir Med. (2021) 9:1407–18. doi: 10.1016/S2213-2600(21)00331-3, PMID: 34480861 PMC8409066

[100] Mohamed-Hussein AAR Aly KME Ibrahim M-EAA. Should aspirin be used for prophylaxis of COVID-19-induced coagulopathy? Med Hypotheses. (2020) 144:109975. doi: 10.1016/j.mehy.2020.109975, PMID: 32531536 PMC7834233

[101] Moncion K Rodrigues L Mackay-Lyons M Eng JJ Billinger SA Ploughman M. Exercise-based stroke rehabilitation: clinical considerations following the COVID-19 pandemic. Neurorehabil Neural Repair. (2022) 36:175. doi: 10.1177/15459683211054175, PMID: 34711094 PMC8721549

[102] Oxley TJ Mocco J Majidi S Kellner CP Shoirah H Singh IP. Large-vessel stroke as A presenting feature of COVID-19 in the young. N Engl J Med. (2020) 382:E60. doi: 10.1056/NEJMc2009787 32343504 PMC7207073

[103] Poillon G Obadia M Perrin M Savatovsky J Lecler A. Cerebral venous thrombosis associated with COVID-19 infection: causality or coincidence? J Neuroradiol. (2021) 48:121–4. doi: 10.1016/j.neurad.2020.05.003, PMID: 32437707 PMC7211586

[104] Qin C Zhou L Hu Z Yang S Zhang S Chen M. Clinical characteristics and outcomes of COVID-19 patients with a history of stroke in Wuhan, China. Stroke. (2020) 51:2219–23. doi: 10.1161/STROKEAHA.120.030365, PMID: 32466735 PMC7282412

[105] Qureshi AI Abd-Allah F Al-Senani F Aytac E Borhani-Haghighi A Ciccone A. Management of acute ischemic stroke in patients with COVID-19 infection: insights from an international panel. Am J Emerg Med. (2020) 38:1548.E5–7. doi: 10.1016/j.ajem.2020.05.018 PMC7211609 32444298

[106] Qureshi AI Baskett WI Huang W Shyu D Myers D Raju M. Acute ischemic stroke and COVID-19: an analysis of 27 676 patients. Stroke. (2021) 52:905–12. doi: 10.1161/STROKEAHA.120.031786, PMID: 33535779 PMC7903982

[107] Rennert RC Wali AR Steinberg JA Santiago-Dieppa DR Olson SE Pannell JS. Epidemiology, natural history, and clinical presentation of large vessel ischemic stroke. Neurosurgery. (2019) 85:S4–8. doi: 10.1093/neuros/nyz042, PMID: 31197329 PMC6584910

[108] Sangalli D Polonia V Colombo D Mantero V Filizzolo M Scaccabarozzi C. A single-centre experience of intravenous thrombolysis for stroke in COVID-19 patients. Neurol Sci. (2020) 41:2325–9. doi: 10.1007/s10072-020-04591-3, PMID: 32656711 PMC7354364

[109] Sasanejad P Afshar Hezarkhani L Arsang-Jang S Tsivgoulis G Ghoreishi A Barlinn K. Safety and outcomes of intravenous thrombolytic therapy in ischemic stroke patients with COVID-19: Cascade initiative. J Stroke Cerebrovasc Dis. (2021) 30:106121. doi: 10.1016/j.jstrokecerebrovasdis.2021.106121, PMID: 34601242 PMC8450304

[110] Shahjouei S Tsivgoulis G Farahmand G Koza E Mowla A Vafaei Sadr A. SARS-CoV-2 and stroke characteristics: a report from the multinational COVID-19 stroke study group. Stroke. (2021) 52:E117–30. doi: 10.1161/STROKEAHA.120.032927 33878892 PMC8078130

[111] Shakibajahromi B Borhani-Haghighi A Haseli S Mowla A. Cerebral venous sinus thrombosis might be under-diagnosed in the COVID-19 era. Eneurologicalsci. (2020) 20:100256. doi: 10.1016/j.ensci.2020.100256, PMID: 32704578 PMC7361048

[112] Spence JD. Management of patients with embolic stroke of unknown source: interpreting the evidence in the light of clinical judgement. Curr Neurol Neurosci Rep. (2022) 22:389–93. doi: 10.1007/s11910-022-01202-w, PMID: 35524921

[113] Steckelings UM Sumners C. Correcting the imbalanced protective Ras in COVID-19 with angiotensin At2-receptor agonists. Clin Sci (Lond). (2020) 134:2987–3006. doi: 10.1042/CS20200922 33210709

[114] Tiedt S Bode FJ Uphaus T Alegiani A Gröschel K Petzold GC. Impact of the COVID-19-pandemic on thrombectomy services in Germany. Neurol Res Pract. (2020) 2:44. doi: 10.1186/s42466-020-00090-0 33251485 PMC7680659

[115] Tornling G Batta R Porter JC Williams B Bengtsson T Parmar K. Seven days treatment with the angiotensin ii type 2 receptor agonist C21 in hospitalized COVID-19 patients; A placebo-controlled randomised multi-Centre double-blind phase 2 trial. Eclinicalmedicine. (2021) 41:101152. doi: 10.1016/j.eclinm.2021.101152, PMID: 34723163 PMC8542174

[116] Xiong Y Wakhloo AK Fisher M. Advances in acute ischemic stroke therapy. Circ Res. (2022) 130:1230–51. doi: 10.1161/CIRCRESAHA.121.319948, PMID: 35420919

[117] Yamamoto M Matsuyama S Li X Takeda M Kawaguchi Y Inoue J-I. Identification of Nafamostat as a potent inhibitor of Middle East respiratory syndrome coronavirus S protein-mediated membrane fusion using the Split-protein-based cell-cell fusion assay. Antimicrob Agents Chemother. (2016) 60:6532–9. doi: 10.1128/AAC.01043-16, PMID: 27550352 PMC5075056

[118] Yang B Wang T Chen J Chen Y Wang Y Gao P. Impact of the COVID-19 pandemic on the process and outcome of thrombectomy for acute ischemic stroke. J Neurointervent Surg. (2020) 12:664–8. doi: 10.1136/neurintsurg-2020-016177, PMID: 32451358

[119] Yang S Yao W Siegler JE Mofatteh M Wellington J Wu J. Shortening door-to-puncture time and improving patient outcome with workflow optimization in patients with acute ischemic stroke associated with large vessel occlusion. BMC Emerg Med. (2022) 22:136. doi: 10.1186/s12873-022-00692-8 35883030 PMC9315077

[120] Yokota S Miyamae T Kuroiwa Y Nishioka K. Novel coronavirus disease 2019 (COVID-19) and cytokine storms for more effective treatments from an inflammatory pathophysiology. J Clin Med. (2021) 10:801. doi: 10.3390/jcm10040801 33671159 PMC7922214

[121] Zhou Y Hong C Chang J Xia Y Jin H Li Y. Intravenous thrombolysis for acute Ischaemic stroke during COVID-19 pandemic in Wuhan, China: a multicentre, retrospective cohort study. J Neurol Neurosurg Psychiatry. (2021) 92:226–8. doi: 10.1136/jnnp-2020-324014, PMID: 33115934

